# Associations Between Social Media Use and Anxiety and Depression Among Older Adults : Cross-Sectional Study

**DOI:** 10.2196/71712

**Published:** 2025-11-10

**Authors:** Jiaoling Huang, Zhenxing Ge, Yijing Chu, Yuge Yan, Wei Zhang, Hong Liang, Yuqi Yang, Hui Wang

**Affiliations:** 1 School of Public Health, Shanghai Jiao Tong University School of Medicine, 227 Chongqing South Road, Shanghai, 200025, China, 86 021-63846590; 2 School of Social Development and Public Policy, Fudan University, Shanghai, China; 3 Shanghai Institute of Infectious Disease and Biosecurity, Shanghai, China

**Keywords:** social media use, addiction, older adults, mental health, anxiety, depression

## Abstract

**Background:**

Social media engagement among older adults has surged worldwide, with China’s older users exceeding 120 million in 2023. However, research remains disproportionately focused on youth. Critically, the dose-response relationship between use intensity and mental health in this population is poorly quantified, especially in rapidly aging societies such as China, where 23% of the population will be aged ≥65 years by 2035.

**Objective:**

This study aimed to outline the social media use status among retired older adults and explore the association between social media use, including time spent on social media and social media addiction, and mental health status.

**Methods:**

A cross-sectional survey was conducted in Shanghai, China, in 2024. A total of 15,986 retired participants were recruited via universities for older adults and primary health care institutions. Short versions of anxiety (the 2-item Generalized Anxiety Disorder scale) and depression (the 2-item Patient Health Questionnaire) scales were used to minimize the required time to complete the questionnaires for older adults. Logistic regressions were used to examine the associations between social media use and mental health after controlling for covariates. Subgroup analysis was conducted considering sex, age, marital status, urbanicity, and socioeconomic status.

**Results:**

The participants had an average age of 68.49 (SD 7.6) years, with most (13,854/15,986, 86.7%) being married and living with their spouse and approximately half (8155/15,986, 51.0%) being male. Our research indicated that over 98% of retired older individuals (15,807/15,986, 98.88%) had used social media, with WeChat, Douyin, and Kuaishou being the most common platforms. Among them, 52.3% (8361/15,986) spent 2 to 3 hours a day on social media, 32.29% (5162/15,986) spent >4 hours a day, and 20.34% (3253/15,986) were addicted to social media. Older adults with ≥6 hours of daily social media use time exhibited higher rates of anxiety (odds ratio [OR] 1.44, 95% CI 1.20-1.72; *P*<.001) and depression (OR 1.50, 95% CI 1.25-1.79; *P*<.001) compared with those who used social media for ≤1 hour per day. Older adults addicted to social media had higher odds of anxiety (OR 2.81, 95% CI 2.57-3.08; *P*<.001) and depression (OR 2.51, 95% CI 2.30-2.75; *P*<.001). Subgroup analyses revealed stronger associations for women, people aged 49-75 years, those with a lower educational level and income, urban residents, and non–solo dwellers.

**Conclusions:**

Retired older adults in Shanghai are an active group of social media users. Using social media for over 6 hours a day and social media addiction were significantly associated with anxiety and depression. Future social media research should pay more attention to older adults and explore these longitudinal relationships.

## Introduction

Mental disorders are increasingly recognized as leading causes of disease burden, with anxiety and depression being the most widespread [1]. The Global Burden of Disease Study (GBD) 2019 Mental Disorders Collaborators reported that, between 1990 and 2019, the global number of disability-adjusted life years due to mental disorders increased from 80.8 million to 125.3 million [2]. In addition to focusing on children and adolescents [3], current studies have focused extensively on older adults due to the recent COVID-19 pandemic. Existing studies suggest that long-term isolation, loneliness, fear of death, and other factors have exacerbated the incidence of mental disorders among older adults [4,5]. At present, we do not have a clear picture of the global prevalence of mental disorders among older adults after the COVID-19 pandemic. However, the latest GBD data show that approximately 14% of adults aged ≥60 years lived with a mental disorder in 2021 [6], which might be underestimated as there has been an ongoing debate about the classification of mental disorders in the GBD and documented diagnostic gaps in older populations [7-9].

Due to the widespread use of electronic devices such as smartphones and tablets, social media is being used in China on an unprecedented scale. By the end of 2023, the number of internet users had reached 1.092 billion, whereas the number of smartphone owners had risen to 974.6 million [10,11]. Critically, older adults represent China’s fastest-growing internet cohort, with users aged ≥60 years increasing by 300% since 2019 [12]. In China, social media platforms such as WeChat, Weibo, Douyin, and Xiaohongshu have gained widespread popularity across the country. The 53rd Statistical Report on the Development of the Internet in China by the China Internet Network Information Center shows that, as of the end of 2023, the size of the internet user group in China aged ≥60 years has reached 170 million (accounting for 57% of the older adult population), with the number of older adults spending more than 10 hours online per day exceeding 100,000 [13]. With the ongoing aging of China’s population, the number of older individuals using social media is increasing annually. At present, although we lack specific data on internet use differences among various older adult age groups, current studies suggest that active older adults, particularly those who have recently retired, are likely to be the primary users of social media [14].

It would be quite interesting to observe how social media use affects the mental health of older adults in China. However, most published studies have focused on social media use among children, students, adolescents, and young adults, and older populations remain relatively understudied compared to these younger groups [15-17]. Although recent years have seen growing research interest in older adults’ digital engagement, several research gaps remain. First, much of the current literature still assumes that older adults are largely averse to using the internet, thereby potentially underestimating their actual engagement with social media today [12]. However, up-to-date surveys on older adults’ social media use are lacking, leaving us without solid evidence of their current patterns of engagement, particularly among active retirees. Second, current research suggests that online social networking has a positive impact on older adults, but recent systematic reviews highlight contradictory findings due to heterogeneous methodologies [18,19]. Reviewing these studies, we found that their measurement of social media use often relied on a simple binary variable—whether the respondent used social media at all. In measuring social media use among older adults, aspects such as frequency, intensity, and even potential addiction should not be overlooked. Third, other research findings in this area are inconclusive, especially because of a relative lack of research conducted on how sex, socioeconomic status (SES), and digital literacy mediate these effects [20]. Thus, this study focused on the vibrant older adult demographic that is most engaged with social media. We aimed to scrutinize the correlation between their social media use (use duration and addiction status) and mental well-being while also examining how this relationship varies among different sexes, age brackets, and SESs.

## Methods

### Participants and Study Design

This cross-sectional study was conducted in Shanghai, a typical Chinese mega city, in 2024, aiming to assess social media use and its association with mental well-being among retired older adults. This study was conducted in 2 phases using convenience sampling. In the first phase, the questionnaire was distributed through universities for older adults in Shanghai. In the second phase, after conducting a preliminary analysis of the questionnaires collected in the first stage, the areas with a smaller number of collected questionnaires were identified. Questionnaires were distributed through primary health care institutions, namely, community health service centers (CHSCs), to areas that either were not initially covered or had a smaller number of responses. All the questionnaires were distributed online. The inclusion criteria were as follows: (1) being retired, (2) no terminal illnesses, (3) ability to speak and read Chinese, and (4) no severe concurrent psychological or psychiatric diseases or other physical diseases that impact physical activities or mental health (self-report diagnosis). The exclusion criteria were as follows: (1) severe cognitive impairment, (2) active psychiatric disorders, and (3) inability to use a smartphone independently. A total of 15,986 valid online questionnaires were collected, and the questionnaire response rate was 99.95% (15,986/15,994).

### Criteria for the Anxiety and Depression Scales

Short versions of anxiety and depression scales were used to minimize the time required to complete the questionnaires for older adults. The 2-item Generalized Anxiety Disorder scale (GAD-2) and the 2-item Patient Health Questionnaire (PHQ-2) were administered. The GAD-2 and PHQ-2 each consist of 2 questions, with each question scored from 0 to 3. The total possible score for each scale is 6 points, with a score of 2 or higher indicating clinically significant symptoms warranting further diagnostic evaluation [21,22]. While we acknowledge that they have a lower sensitivity than that of the full scales (the 9-item Patient Health Questionnaire and 7-item Generalized Anxiety Disorder scale), these instruments demonstrate adequate validity for population-level screening in older adults (area under the curve=0.78-0.84), particularly given the optimized acceptability in older cohorts with cognitive fatigue concerns [23-25].

### Social Media Use

Social media use was represented using 2 variables: time spent on social media and social media addiction. Time spent on social media was measured using the self-reported amount of time spent on social media each day, categorized into 4 groups: ≤1 hour, 2 to 3 hours, 4 to 5 hours, and ≥6 hours. Social media addiction was assessed using the Bergen Social Media Addiction Scale, with a cutoff score of 19. A score above 19 indicates addiction to social media [26].

### Covariates

The covariates in this study included sex (male=1; female=2), age, educational level (senior high school and lower=1; junior college=2; university and higher=3), marital status (married and living with spouse=1; married but not living with spouse=2; divorced or widowed=3; unmarried=4), urban or rural residence, living status (living alone=1; not living alone=2), and monthly income (≤¥6000 [US $842.05]=1; ¥6001‐¥9000 [US $842.19-$1263.07]=2; ≥¥9001 [US $1263.21]=3) of the active older individuals. Health behavior–related variables encompassed physical activity (regular exercise=1; no regular exercise=2), sedentary hours (<0.5=1; 0.5‐4=2; >4=3), sleep duration (≤6 hours=1; 7‐8 hours=2; ≥9 hours=3), and social engagement levels (hardly ever socialize=1; sometimes socialize=2; often socialize=3). All the covariates were based on the self-reports of the research subjects.

### Statistical Analyses

For the descriptive statistics, we used the mean (with SE) for continuous variables and the number (with weighted percentage) for categorical variables. To identify association factors for anxiety and depression in older adults, we conducted logistic regression to calculate odds ratios (ORs) with 95% CIs. Adjusted ORs were obtained through multivariate regression analyses, using selection methods to account for potential confounding variables. Subgroup analyses were conducted to explore the associations between social media use and mental health among individuals of different sexes, ages, educational levels, marital statuses, monthly incomes, urban and rural residences, and living conditions. Database management and all statistical analyses were conducted using the *Survey* package in R (version 4.3.1; R Foundation for Statistical Computing) and ArcMap (version 10.8; Esri), which are software for statistical computing and plotting, respectively. A 2-sided *P* value of <.05 was considered statistically significant.

### Ethical Considerations

This study was approved by the Ethics Committee of Public Health and Nursing Research, Shanghai Jiao Tong University School of Medicine (approval: SJUPN-2024-041-KS1). Written informed consent was obtained from all participants, with procedures for illiterate subjects involving witnessed verbal consent documented using institutional review board–approved forms. For secondary data analyses, original consent permitted anonymized reuse. All data were deidentified through replacement of personal identifiers with unique codes and aggregated for analysis to ensure confidentiality. There was no charge for participating in the study, and no compensation was provided to the participants. No identifiable images or personal information appear in this manuscript or the supplementary materials. This study was conducted in accordance with the Declaration of Helsinki.

## Results

### Descriptive Analysis

Table 1 shows the descriptive statistics of the sample. The average age of the respondents was 68.49 (SD 7.6) years. Men constituted 51.0% (8155/15,986) of the sample. A significant portion (5633/15,986, 35.2%) had received education at the university level or higher. Most respondents (13,854/15,986, 86.7%) were married and living with their spouse. A substantial number (9669/15,986, 60.5%) had a monthly income exceeding ¥9000 (US $1263.07), and 67.8% (10,833/15,986) resided in urban areas. The prevalence rates of anxiety and depression were 20.1% (3218/15,986) and 21.7% (3467/15,986), respectively. Regarding daily social media use time, 15.4% (2463/15,986) spent no more than 1 hour, 52.3% (8361/15,986) spent between 2 and 3 hours, 25.9% (4134/15,986) spent between 4 and 5 hours, and 6.4% (1028/15,986) spent more than 6 hours. The prevalence of social media addiction was 20.3% (3253/15,986). The social media platforms most used by older adults were WeChat, Douyin, and Kuaishou (Multimedia Appendix 1), and the most viewed type of content was current news and health-related information (Multimedia Appendix 1).

**Table 1.** Baseline sociodemographic data (N=15,986).

Characteristic	Values
Sex, n (%)	
Male	8155 (51.0)
Female	7831 (49.0)
Age (y), mean (SD)	68.49 (7.6)
Educational level, n (%)	
Senior high school and lower	3948 (24.7)
Junior college	6405 (40.1)
University and higher	5633 (35.2)
Marital status, n (%)	
Married and living with spouse	13,854 (86.7)
Married but not living with spouse	356 (2.2)
Divorced or widowed	1675 (10.5)
Unmarried	101 (0.6)
Monthly income, n (%)	
≤¥6000 (US $842.05)	2922 (18.3)
¥6001-¥9000 (US $842.19-$1263.07)	3395 (21.2)
≥¥9001 (US $1263.21)	9669 (60.5)
Urban or rural residence, n (%)	
Urban	10,833 (67.8)
Rural	5153 (32.2)
Social media use (hours per day), n (%)	
≤1	2463 (15.4)
2-3	8361 (52.3)
4-5	4134 (25.9)
≥6	1028 (6.4)
Social media addiction, n (%)	
No	12,733 (79.7)
Yes	3253 (20.3)
Anxiety, n (%)	
No	12,768 (79.9)
Yes	3218 (20.1)
Depression, n (%)	
No	12,519 (78.3)
Yes	3467 (21.7)

### Logistic Analyses

Among the older adults with longer social media use times, a higher proportion exhibited higher scores for anxiety and depression (284/1028, 27.6% and 304/1028, 29.6%, respectively). Compared to those who used social media for 1 hour or less per day, those who used social media for 6 hours or more per day had 1.44 times the odds of anxiety (*P*<.001) and 1.50 times the odds of depression (*P*<.001). Among the older adults with social media addiction, a higher proportion had higher scores for anxiety and depression (1140/3,253, 35.0% and 1146/3253, 35.2%, respectively). The odds of anxiety were 2.81 times higher in those with addiction versus those without (*P*<.001), whereas their odds of depression were 2.51 times higher (*P*<.001; Table 2)

**Table 2.** Associations between social media use and mental health (N=15,986).

Characteristic	Participants, n (%)		Unadjusted analysis		Adjusted analysis^a^	
	No anxiety	Anxiety	OR^b^ (95% CI)	*P* value	OR (95% CI)	*P* value
Anxiety						
Social media use time (h per d)						
≤1 (n=2463)	2014 (81.8)	449 (18.2)	—^c^	—	—	—
2-3 (n=8361)	6728 (80.5)	1633 (19.5)	1.09 (0.97-1.22)	.15	1.16 (1.03-1.31)	.01
4-5 (n=4134)	3282 (79.4)	852 (20.6)	1.16 (1.03-1.32)	.02	1.17 (1.02-1.34)	.02
≥6 (n=1028)	744 (72.4)	284 (27.6)	1.71 (1.44-2.03)	<.001	1.44 (1.20-1.72)	<.001
Social media addiction						
No (n=12,733)	10,655 (83.7)	2078 (16.3)	—	—	—	—
Yes (n=3253)	2113 (65.0)	1140 (35.0)	2.77 (2.54-3.01)	<.001	2.81 (2.57-3.08)	<.001
Depression						
Social media use time (hours per day)						
≤1 (n=2463)	1988 (80.7)	475 (19.3)	—	—	—	—
2-3 (n=8361)	6624 (79.2)	1737 (20.8)	1.10 (0.98-1.23)	.11	1.20 (1.07-1.35)	.002
4-5 (n=4134)	3183 (77.0)	951 (23.0)	1.25 (1.11-1.41)	<.001	1.29 (1.14-1.48)	<.001
≥6 (n=1028)	724 (70.4)	304 (29.6)	1.76 (1.49-2.08)	<.001	1.50 (1.25-1.79)	<.001
Social media addiction						
No (n=12,733)	10,412 (81.8)	2321 (18.2)	—	—	—	—
Yes (n=3253)	2107 (64.8)	1146 (35.2)	2.44 (2.24-2.75)	<.001	2.51 (2.30-2.75)	<.001

a Adjusted model: age, educational level, marital status, urban or rural residence, living status, monthly income, physical activity, sedentary time, sleep duration, and social engagement levels.

b OR: odds ratio.

c Reference group.

### Subgroup Analyses

#### Associations Between Time Spent on Social Media and Mental Health

**Figure 1.**
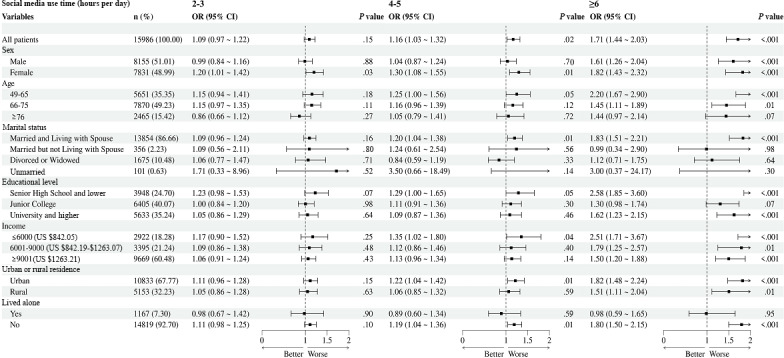
Subgroup analysis of the associations between social media use time and anxiety. OR: odds ratio.

In the subgroup analysis of social media use time and anxiety (Figure 1), among the older adults who used social media for 6 or more hours per day, the odds of anxiety were higher for women (OR 1.82, 95% CI 1.43-2.32 vs OR 1.61, 95% CI 1.26-2.04; Table S1 in Multimedia Appendix 1), those aged 49 to 65 years (OR 2.20, 95% CI 1.67-2.90 vs OR 1.45, 95% CI 1.11-1.89; Table S2 in Multimedia Appendix 1), individuals who were married and living with their spouse (OR 1.83, 95% CI 1.51-2.21 vs OR 0.99, 95% CI 0.34-2.90; Table S3 in Multimedia Appendix 1), individuals with lower levels of education (OR 2.58, 95% CI 1.85-3.60 vs OR 1.62, 95% CI 1.23-2.15; Table S4 in Multimedia Appendix 1), those with a lower monthly income (OR 2.51, 95% CI 1.781 3.67 vs OR 1.79, 95% CI 1.25-2.57; Table S5 in Multimedia Appendix 1), those residing in urban areas (OR 1.82, 95% CI 1.48-2.24 vs OR 1.51, 1.11-2.04; Table S6 in Multimedia Appendix 1), and those not living alone (OR 1.80, 95% CI 1.50-2.15; Table S7 in Multimedia Appendix 1).

**Figure 2.**
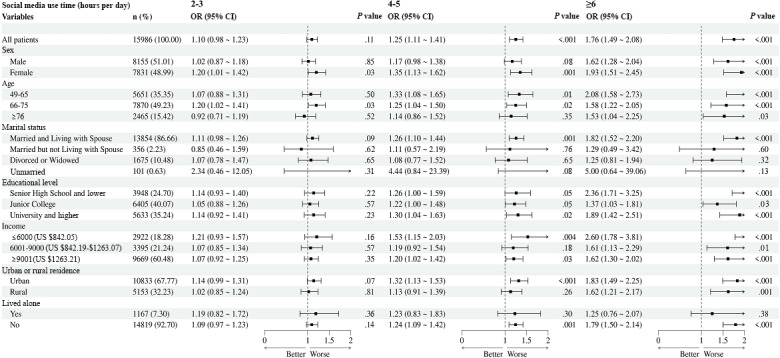
Subgroup analysis of the associations between social media use time and depression. OR: odds ratio.

In the subgroup analysis of social media use time and depression (Figure 2), among the older adults who used social media for 6 or more hours per day, the odds of depression were higher for women (OR 1.93, 95% CI 1.51-2.45 vs OR 1.62, 95% CI 1.28-2.04; Table S1 in Multimedia Appendix 1), those aged 49 to 65 years (OR 2.08, 95% CI 1.58-2.73 vs OR 1.58, 95% CI 1.22-2.05; Table S2 in Multimedia Appendix 1Appendix 4), individuals who were married and living with their spouse (OR 1.82, 95% CI 1.52-2.20 vs OR 1.25, 95% CI 0.81-1.94; Table S3 in Multimedia Appendix 1), individuals with lower levels of education (OR 2.36, 95% CI 1.71-3.25 vs OR 1.89, 95% CI 1.42-2.51; Table S4 in Multimedia Appendix 1), those with a lower monthly income (OR 2.60, 95% CI 1.78-3.81 vs OR 1.62, 95% CI 1.30-2.02; Table S5 in Multimedia Appendix 1), those residing in urban areas (OR 1.83, 95% CI 1.49-2.25 vs OR 1.62, 95% CI 1.21-2.17; Table S6 in Multimedia Appendix 1), and those not living alone (OR 1.79, 95% CI 1.50-2.14; Table S7 in Multimedia Appendix 1).

#### Associations Between Social Media Addiction and Mental Health

**Figure 3.**
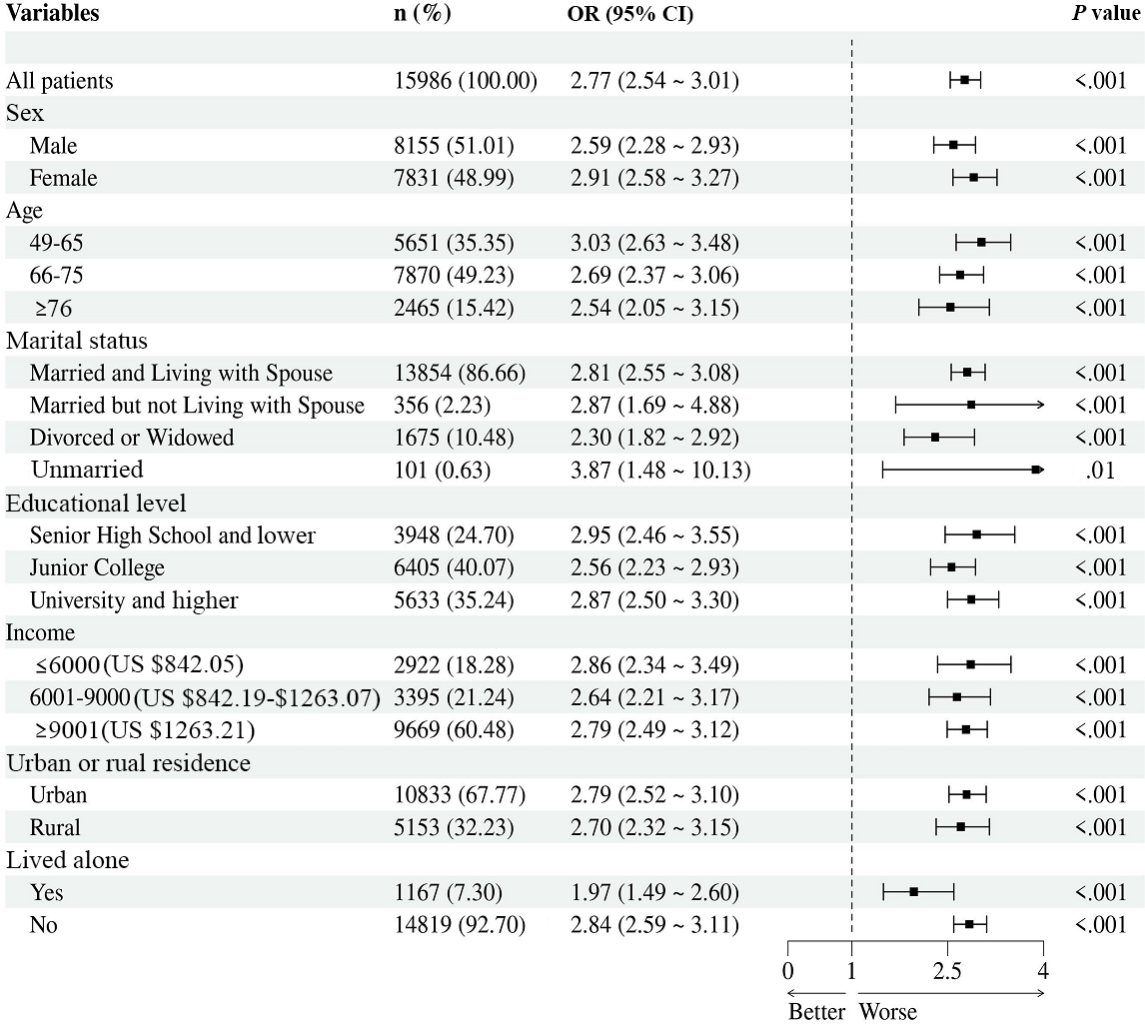
Subgroup analysis of the associations between social media addiction and anxiety. OR: odds ratio.

In the subgroup analysis of social media addiction and anxiety (Figure 3), among the older adults who were addicted to social media, the odds of anxiety were higher for women (OR 2.91, 95% CI 2.58-3.27 vs OR 2.59, 95% CI 2.28-2.93; Table S1 in Multimedia Appendix 1), those aged 49 to 65 years (OR 3.03, 95% CI 2.63-3.28 vs OR 2.69, 95% CI 2.37-3.06; Table S2 in Multimedia Appendix 1), individuals who were single (unmarried; OR 3.87, 95% CI 1.48-10.13 vs OR 2.87, 95% CI 1.69-4.88; Table S3 in Multimedia Appendix 1), those with lower levels of education (OR 2.95, 95% CI 2.46-3.55 vs OR 2.87, 95% CI 2.50-3.30; Table S4 in Multimedia Appendix 1), those with a lower monthly income (OR 2.86, 95% CI 2.34-3.49 vs OR 2.79, 95% CI 2.49-3.12; Table S5 in Multimedia Appendix 1), those residing in urban areas (OR 2.79, 95% CI 2.52-3.10 vs OR 2.70, 95% CI 2.32-3.15; Table S6 in Multimedia Appendix 1), and those not living alone (OR 2.84, 95% CI 2.59-3.11 vs OR 1.97, 95% CI 1.49-2.60; Table S7 in Multimedia Appendix 1).

**Figure 4.**
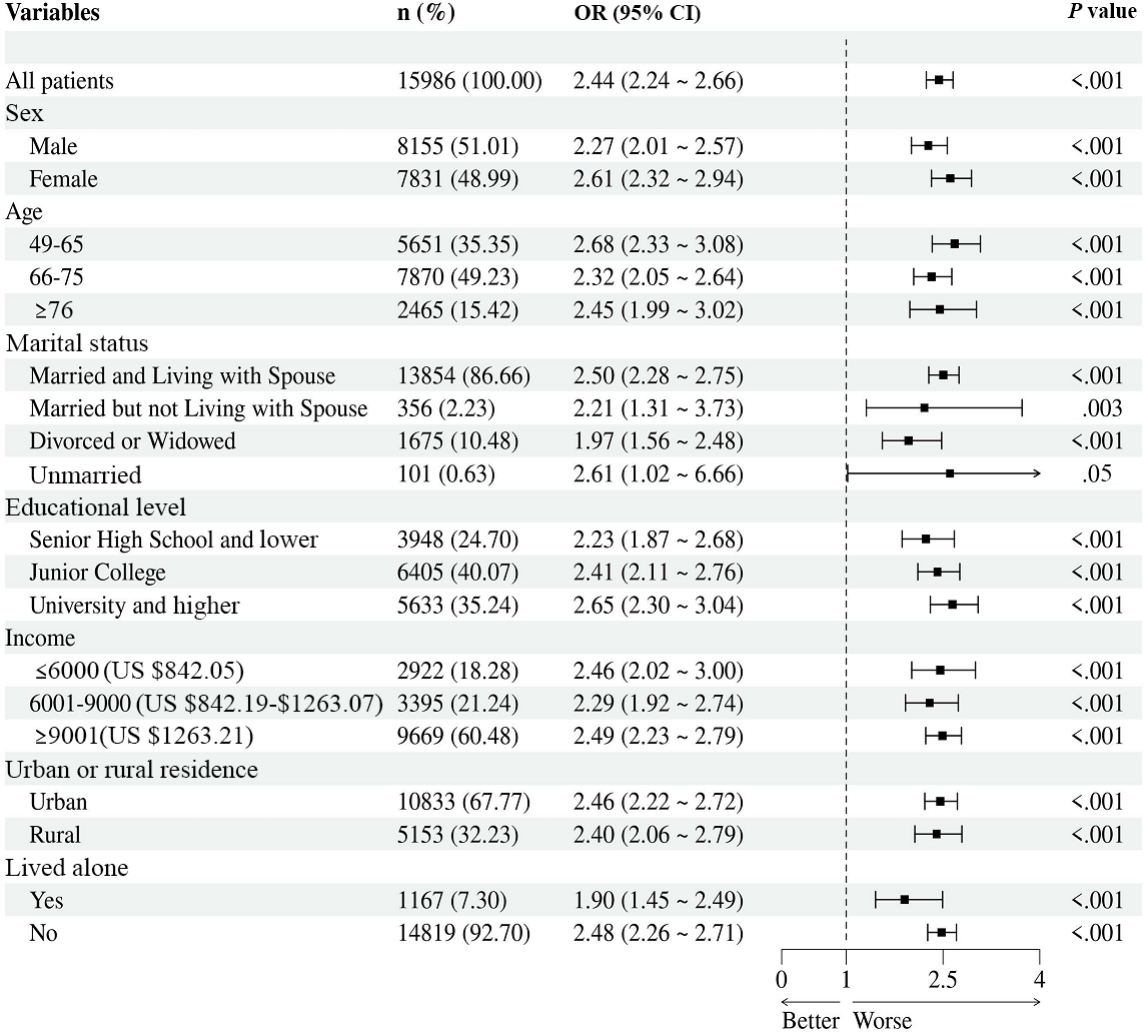
Subgroup analysis of the associations between social media addiction and depression. OR: odds ratio.

Social media addiction posed slightly higher odds of anxiety. In the subgroup analysis of depression and social media addiction (Figure 4), among the older adults who were addicted to social media, the odds of anxiety were higher for women (OR 2.61, 5%CI 2.32-2.94 vs OR 2.27, 95% CI 2.01-2.57; Table S1 in Multimedia Appendix 1), those aged 49 to 65 years (OR 2.68, 95% CI 2.33-3.08 vs OR 2.45, 95% CI 1.99-3.02; Table S2 in Multimedia Appendix 1), individuals who were single (unmarried; OR 2.61, 95% CI 1.02-6.66 vs OR 2.50, 95% CI 2.28-2.75; Table S3 in Multimedia Appendix 1), those with higher levels of education (OR 2.65, 95% CI 2.30-3.04 vs OR 2.41, 95% CI 2.11-2.76; Table S4 in Multimedia Appendix 1), those with a higher monthly income (OR 2.49, 95% CI 2.23-2.79 vs OR 2.46, 95% CI 2.02-3.00; Table S5 in Multimedia Appendix 1), those residing in urban areas (OR 2.46, 95% CI 2.22-2.72 vs OR 2.40, 95% CI 2.06-2.79; Table S6 in Multimedia Appendix 1), and those not living alone (OR 2.48, 95% CI 2.26-2.71 vs OR 1.90, 95% CI 1.45-2.49; Table S7 in Multimedia Appendix 1).

## Discussion

### Principal Findings

We observed that, in Shanghai, a typical Chinese megacity, older adults seem to have entered a new digital era. Our research found that over 98% of retired older adults in Shanghai (15,807/15,986, 98.8%) had ever used social media, particularly platforms such as WeChat, Douyin, and Kuaishou, with over 30% of them (5162/15,986, 32.3%) using it for more than 4 hours a day and over 20% (3253/15,986, 20.4%) being addicted to it. The frequency of social media use and the addiction rate among older adults in Shanghai have reached or even surpassed those of young people. Riehm et al [27] reported that approximately 80% of adolescents use social media for no more than 3 hours. In a study conducted by Eichenberg et al [28], approximately 20% of students were identified as being addicted to social media. On buses or subways in China, an increasing number of older adults being “phubbers” (people who are constantly looking at their phones) can be observed, mainly browsing short videos and social media feeds such as Moments on WeChat. Current social media platforms offer a variety of social features for older adults, including adding new friends and creating social circles, having voice and video conversations, sharing and commenting on daily life, and browsing and interacting with short videos. However, Jin et al [29] pointed out that more older adults are using social media such as WeChat and short videos, which can affect their daily life and mental health, possibly leading to addiction.

In our study, a positive correlation was found indicating that longer durations of social media use were associated with increased anxiety. Specifically, we found that social media use duration became highly associated with anxiety once it exceeded 6 hours, and such difference was not observed among those who used social media for less than 1 hour, 2 to 3 hours, or 4 to 5 hours. Similarly, the relationship between time spent on social media and depression was also positive and more prominent than that for anxiety. Existing research, which is limited, has provided contradictory evidence. Yan et al [30] investigated the association between internet exclusion and depressive symptoms among older adults in 32 countries and found that internet use was negatively associated with depressive symptoms among older adults. Due to limitations in public databases, the aforementioned study only examined the relationship between internet use (yes or no) and depression among older adults, not the duration of social media use or social media addiction. In contrast, our analytic framework aligns with a growing body of evidence that documents a positive association between social media use frequency and intensity and depressive symptoms in later life [31,32]. Two factors appear to underlie this positive association among “young-old” retirees. First, active, healthy older adults increasingly replace offline social, physical, and community engagements with prolonged online activity, eroding face-to-face support networks and diminishing the mood-regulating benefits of in-person interaction. Second, older users predominantly engage in passive content consumption rather than active creation, resulting in frequent upward social comparisons and algorithm-amplified exposure to negative information—such as health-related rumors and sensationalized news [33]. These passive, comparison-driven experiences exacerbate feelings of inadequacy, anxiety, and hopelessness, thereby translating extended daily social media use into heightened depressive and anxiety symptomatology.

We attempted to explain the negative impact of social media on the mental health of older adults, and we infer that it may be related to their use habits. When older adults use social media, they often scroll and browse, such as watching short videos on platforms such as Douyin or scrolling through their Moments feed on the WeChat app, which is classified as passive social media use. According to Verduyn et al [34], passive social media use is defined as monitoring or observing content and behavior on social media platforms without direct exchanges with others (eg, scrolling and browsing), whereas active use involves more direct interactions such as posting, commenting, and sending private messages [35]. A substantial body of research has revealed a positive relationship between passive social media use and anxiety or depression among younger individuals [36]. Passive social media use is considered a cause of stress and anxiety, largely due to upward social comparison triggered by prevailing positive self-presentations [37,34]. In addition, the older adult group involved in this study was in the process of entering old age, with many having just started retirement or experiencing the empty nest stage. This cohort of older adults is unique in that they are part of the internet era, being familiar with smartphones and internet browsing. Spending a long time on social media tends to decrease their opportunities for face-to-face communication with family and friends, and passive use of social media further limits their chances to express themselves and engage in interactive communication. Further research should be conducted to delve into the pathways and mechanisms through which prolonged social media use among these younger members of the older population leads to anxiety and depression.

Additionally, our study provides clear evidence of a strong association between social media addiction and mental health issues among older adults. The odds of anxiety and depression for older individuals with social media addiction were 2.80 and 2.51 times higher, respectively, than for those without addiction (*P*<.001). This positive correlation has been consistently found in existing studies targeting adolescent populations [38,39]. Relatively few studies have focused on social media addiction among older adults; however, the results consistently show that social media addiction is highly correlated with mental health problems [40]. Some studies also suggest that mental health problems may lead to social media addiction among older adults [41]. Further exploration is needed to understand the causal relationship between the two.

Our study further identified that female, younger, married, and non–solo-dwelling older individuals are more likely to experience anxiety and depression when using social media. Sex and age factors have been partially explained in existing literature involving adolescents, such as female and younger individuals being more sensitive and active [40]. Özbek and Karaş [41] pointed out that sex roles may restrict women socially, so women may prefer to use social media to meet their social needs at home. Older individuals who are married and living with family or partners have also been identified (this was derived from 2 questions in the questionnaire. One was about marital status, and the other was about who one currently lives with, suggesting that, even when surrounded by others, they may still be at risk because social media can supplant direct human interaction, which in turn can be detrimental to their mental well-being [41]. In addition, we found that the association between social media use and mental well-being varied significantly by SES, with anxiety and depression being more prevalent among individuals with lower SES (lower income and educational level). Wang et al [42] identified area-level SES as a moderator between internet use and depressive symptoms, with an explanation that individuals residing in lower-SES regions were constrained by limited resources and social support, which could exacerbate the association between internet use and depressive symptoms. However, we also noticed that highly educated older adults who were addicted to social media were more likely to experience depression. We believe that the causal relationship requires further investigation. In addition, the mental health of older people in cities is more susceptible to the influence of social media, which is consistent with previous studies. Xu and Zhang [43] believe that although urban older people have easier access to the internet, information overload, online social comparison, and substitution of offline social interaction might be the underlying causes of higher levels of depression.

Drawing on our findings, we propose the following evidence-based policy recommendations. First, regulatory authorities should prioritize the development of age-friendly digital ecosystems by mandating algorithmic reforms that promote health-related content and enforce periodic use break reminders. This addresses our critical finding that older adults’ social media engagement has reached levels comparable to those of younger users. Second, community health services should implement offline alternative programs such as subsidized “screen-free hour” activities and intergenerational interaction hubs to mitigate the mental health risks associated with prolonged screen time. Finally, resource allocation should be precisely targeted toward the high-risk subgroups identified in our stratified analyses—specifically, urban women aged 60 to 74 years, individuals with low SES, and non–solo dwellers—through neighborhood-based digital literacy workshops, mental health screenings at CHSCs, and family-mediated device management initiatives. These measures aim to foster a healthier integration of the digital and physical aspects of life for China’s aging population.

### Limitations

Some limitations remain. First, although we targeted the older population, we mainly focused on the relatively younger and more vibrant segment of this demographic as they are the primary group of older social media users. However, we are not clear on the situation of more advanced-age older adults. Critically, we excluded working older people—omitting 8.9% of China’s population aged >65 years, who may experience distinct digital stressors. Meanwhile, despite efforts to balance district-level representation through supplementary CHSC sampling, participants from these 2 sources differed in age and education profiles. Caution is warranted when generalizing the findings to isolated older adult populations beyond educational institutions. Second, self-reported data may incur recall bias, and the GAD-2 and PHQ-2 are screening tools, not clinical diagnoses. Our cutoff scores (≥3) may not generalize across subpopulations. Third, we merely identified the relationship between social media use and mental health, but the causality between the two remains unclear. A cohort study is recommended in the future, which could further clarify this causal relationship. Fourth, the links between social media use and mental health issues are not direct, and various factors such as prolonged sitting, sleep, and social support might play a mediating role, which needs further exploration. Fifth, despite our screening procedures (self-report diagnosis), residual confounding from undiagnosed clinical disorders may persist. Individuals with subclinical anxiety or depression might overuse social media and report poorer mental health, inflating the observed associations. Future studies should incorporate structured clinical interviews to better control for this confounder. Finally, exploratory subgroup analyses did not include formal interaction testing due to sample constraints.

### Conclusions

We focused on retired older adults instead of on adolescents and explored the impact of social media use on their mental well-being, yielding some interesting findings. We found that older adults in Shanghai have a high frequency of social media use, with over 30% of them (15,807/15,986, 32.3%) spending more than 4 hours per day on social media and over 20% (3253/15,986, 20.4%) exhibiting signs of social media addiction. Significant associations between social media use, including time spent on and addiction to social media, and mental health, including anxiety and depression, were identified. Furthermore, subgroup analysis revealed that older individuals with lower SES who were female, younger, married, and not living alone were more prone to experiencing anxiety and depression through their engagement with social media.

## Supplementary material

10.2196/71712 Multimedia Appendix 1 Word clouds of the most used social media platforms and most viewed content, plus full logistic regression and subgroup analysis tables for social media use versus anxiety and depression.

## Abbreviations

CHSC: community health service center
GAD-2: 2-item Generalized Anxiety Disorder scale
GBD: Global Burden of Disease Study
OR: odds ratio
PHQ-2: 2-item Patient Health Questionnaire
SES: socioeconomic status
