# Supplementary material for: Associations Between Social Media Use and Anxiety and Depression Among Older Adults : Cross-Sectional Study
Source: JMIR Aging. 2025 Nov 10;8:e71712. doi: 10.2196/71712 (PMC12603664; doi:10.2196/71712)
Supplement: Multimedia Appendix 1 [file aging-v8-e71712-s001.docx]

**Multimedia Appendix**

**Multimedia Appendix** **1.** The word cloud of the most common social media type used by older adults


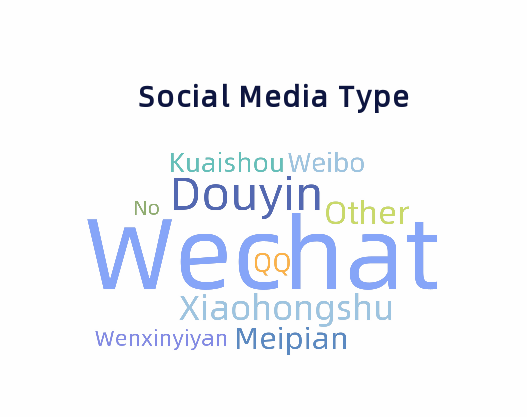


**Multimedia Appendix** **2.** The word cloud of the browsed content what older people view on social media


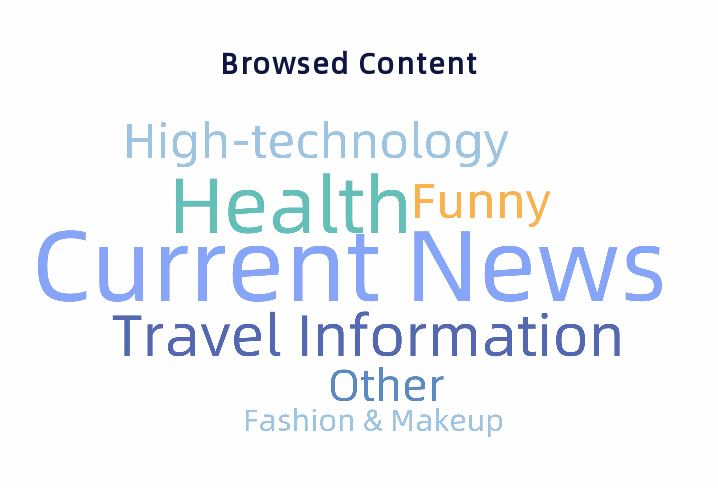


**Multimedia Appendix 3.** Associations between social media usage and mental health grouped by gender

| Variables | n  (N=15986) | Proportion, % | | | Adjusted Analyses | |
| --- | --- | --- | --- | --- | --- | --- |
|  |  | Low | Middle | High | OR (95%CI) | *p* |
| **Anxiety** | | | | | | |
| **Social Media Usage Time (h/d)- Male(N=8155)** | | | | |  |  |
| ≤1 | 1308 | 73.93 | 24.54 | 1.53 | - | - |
| 2-3 | 4279 | 71.11 | 27.55 | 1.33 | 0.99(0.84-1.16) | 0.884 |
| 4-5 | 2055 | 70.36 | 27.59 | 2.04 | 1.04(0.87-1.24) | 0.695 |
| ≥6 | 513 | 61.60 | 33.92 | 4.48 | 1.61(1.26-2.32) | <0.001 |
| **Social Media Addiction** | | | | |  |  |
| No | 6611 | 74.47 | 24.20 | 1.33 | - | - |
| Yes | 1544 | 54.99 | 41.51 | 3.50 | 2.59(2.28-2.93) | <0.001 |
| **Social Media Usage Time (h/d)- Female(N=7831)** | | | | |  | |
| ≤1 | 1155 | 69.965 | 27.71 | 2.34 | - | - |
| 2-3 | 4082 | 64.55 | 33.76 | 1.69 | 1.20(1.01-1.42) | 0.034 |
| 4-5 | 2079 | 61.42 | 37.33 | 1.25 | 1.30(1.08-1.55) | 0.005 |
| ≥6 | 515 | 58.24 | 36.89 | 4.85 | 1.82(1.43-2.32) | <0.001 |
| **Social Media Addiction** | | | | |  |  |
| No | 6122 | 69.14 | 29.39 | 1.47 | - | - |
| Yes | 1709 | 46.05 | 50.61 | 3.34 | 2.91(2.58-3.27) | <0.001 |
| **Depression** | | | | | | |
| **Social Media Usage Time (h/d)- Male(N=8155)** | | | | |  |  |
| ≤1 | 1308 | 67.28 | 30.81 | 1.91 | - | - |
| 2-3 | 4279 | 65.79 | 32.90 | 1.31 | 1.20(1.01-1.42) | 0.033 |
| 4-5 | 2055 | 63.99 | 33.48 | 2.53 | 1.35(1.13-1.62) | 0.001 |
| ≥6 | 513 | 56.72 | 37.82 | 5.46 | 1.93(1.51-2.45) | <0.001 |
| **Social Media Addiction** | | | | |  |  |
| No | 6611 | 68.31 | 30.13 | 1.56 | - | - |
| Yes | 1544 | 50.84 | 45.40 | 3.76 | 2.27(2.01-2.57) | <0.001 |
| **Social Media Usage Time (h/d)-Female(N=7831)** | | | | |  | |
| ≤1 | 1155 | 68.74 | 28.83 | 2.43 | - | - |
| 2-3 | 4082 | 63.81 | 34.62 | 1.57 | 1.33(1.15-1.54) | 0.010 |
| 4-5 | 2079 | 60.85 | 37.85 | 1.30 | 1.41(1.20-1.66) | <0.001 |
| ≥6 | 515 | 54.95 | 41.94 | 3.11 | 1.57(1.25-1.96) | <0.001 |
| **Social Media Addiction** | | | | |  |  |
| No | 6122 | 68.00 | 30.51 | 1.49 | - | - |
| Yes | 1709 | 45.87 | 51.55 | 2.58 | 2.61(2.32-3.94) | <0.001 |

**Multimedia Appendix 4.** Associations between social media usage and mental health grouped by age

| Variables | n  (N=15986) | Proportion, % | | | Adjusted Analyses | |
| --- | --- | --- | --- | --- | --- | --- |
|  |  | Low | Middle | High | OR (95%CI) | *p* |
| **Anxiety** | | | | | | |
| **Social Media Usage Time (h/d)- Age 49-65 (N=5651)** | | | | |  | |
| ≤1 | 837 | 73.36 | 25.33 | 1.31 | - | - |
| 2-3 | 2900 | 68.86 | 29.90 | 1.24 | 1.15(0.94-1.41) | 0.178 |
| 4-5 | 1505 | 65.65 | 32.82 | 1.53 | 1.25(1.00-1.56) | 0.048 |
| ≥6 | 409 | 56.72 | 37.16 | 6.12 | 2.20(1.67-2.90) | <0.001 |
| **Social Media Addiction** | | | | |  |  |
| No | 4342 | 73.40 | 25.43 | 1.17 | - | - |
| Yes | 1309 | 49.20 | 47.44 | 3.36 | 3.03(2.63-3.48) | <0.001 |
| **Social Media Usage Time (h/d)- Age 66-75 (N=7870)** | | | | |  | |
| ≤1 | 1245 | 73.65 | 24.58 | 1.77 | - | - |
| 2-3 | 4157 | 68.87 | 29.73 | 1.40 | 1.15(0.97-1.35) | 0.109 |
| 4-5 | 2016 | 67.51 | 31.15 | 1.34 | 1.16(0.96-1.39) | 0.118 |
| ≥6 | 452 | 64.60 | 32.74 | 2.65 | 1.45(1.11-1.89) | 0.006 |
| **Social Media Addiction** | | | | |  |  |
| No | 6388 | 72.89 | 25.81 | 1.30 | - | - |
| Yes | 1482 | 52.43 | 45.14 | 2.43 | 2.69(2.37-3.06) | <0.001 |
| **Social Media Usage Time (h/d)- Age ≥76 (N=2465)** | | | | |  | |
| ≤1 | 381 | 64.05 | 32.28 | 3.67 | - | - |
| 2-3 | 1304 | 62.73 | 34.82 | 2.45 | 0.86(0.66-1.12) | 0.271 |
| 4-5 | 613 | 61.01 | 36.05 | 2.94 | 1.05(0.79-1.41) | 0.724 |
| ≥6 | 167 | 55.09 | 38.32 | 6.59 | 1.44(0.97-2.14) | 0.074 |
| **Social Media Addiction** | | |  | |  | |
| No | 2003 | 65.55 | 32.25 | 2.20 | - | - |
| Yes | 462 | 46.54 | 46.75 | 6.71 | 2.54(2.05-3.15) | <0.001 |
| **Depression** | | | | | | |
| **Social Media Usage Time (h/d)- Age 49-65 (N=5651)** | | | | |  | |
| ≤1 | 837 | 68.22 | 29.75 | 2.03 | - | - |
| 2-3 | 2900 | 67.07 | 32.03 | 0.90 | 1.07(0.88-1.31) | 0.496 |
| 4-5 | 1505 | 62.66 | 35.35 | 1.99 | 1.33(1.08-1.65) | 0.009 |
| ≥6 | 409 | 53.54 | 41.81 | 4.65 | 2.08(1.58-2.73) | <0.001 |
| **Social Media Addiction** | | | |  |  | |
| No | 4342 | 70.29 | 28.40 | 1.31 | - | - |
| Yes | 1309 | 47.82 | 49.50 | 2.68 | 2.68(2.33-3.08) | <0.001 |
| **Social Media Usage Time (h/d)- Age 66-75 (N=7870)** | | | | |  | |
| ≤1 | 1245 | 70.60 | 27.87 | 1.53 | - | - |
| 2-3 | 4157 | 65.19 | 33.41 | 1.40 | 1.20(1.02-1.41) | 0.032 |
| 4-5 | 2016 | 64.78 | 33.88 | 1.34 | 1.25(1.04-1.50) | 0.017 |
| ≥6 | 452 | 61.28 | 35.84 | 2.88 | 1.58(1.22-2.05) | 0.030 |
| **Social Media Addiction** | | | | |  |  |
| No | 6388 | 69.09 | 29.63 | 1.28 | - | - |
| Yes | 1482 | 51.21 | 46.42 | 2.37 | 2.32(2.05-2.64) | <0.001 |
| **Social Media Usage Time (h/d)- Age ≥76 (N=2465)** | | | | |  | |
| ≤1 | 381 | 58.79 | 36.75 | 4.46 | - | - |
| 2-3 | 1304 | 58.67 | 38.57 | 2.76 | 0.92(0.71-1.19) | 0.343 |
| 4-5 | 613 | 54.00 | 42.41 | 3.59 | 1.14(0.86-1.52) | 0.347 |
| ≥6 | 167 | 46.70 | 46.11 | 7.19 | 1.53(1.04-2.25) | 0.030 |
| **Social Media Addiction** | | | |  |  | |
| No | 2003 | 60.61 | 36.65 | 2.74 | - | - |
| Yes | 462 | 39.82 | 53.25 | 6.93 | 2.54(1.99-3.02) | <0.001 |

**Multimedia Appendix 5**. Associations between social media usage and mental health grouped by marriage

| Variables | n  (N=15986) | Proportion, % | | | Adjusted Analyses | |
| --- | --- | --- | --- | --- | --- | --- |
|  |  | Low | Middle | High | OR (95%CI) | *p* |
| **Anxiety** | | | | | | |
| **Social Media Usage Time (h/d)- Married and Living with Spouse (N=13854)** | | | | |  | |
| ≤1 | 2145 | 73.85 | 24.43 | 1.72 | - | - |
| 2-3 | 7337 | 69.52 | 29.26 | 1.22 | 1.09(0.96-1.24) | 0.163 |
| 4-5 | 3525 | 67.44 | 31.23 | 1.33 | 1.20(1.04-1.38) | 0.012 |
| ≥6 | 847 | 60.68 | 35.54 | 3.78 | 1.83(1.51-2.21) | <0.001 |
| **Social Media Addiction** | | | | |  |  |
| No | 11110 | 73.31 | 25.52 | 1.17 | - | - |
| Yes | 2744 | 52.15 | 45.12 | 2.73 | 2.81(2.55-3.08) | <0.001 |
| **Social Media Usage Time (h/d)- Married and not Living with Spouse (N=356)** | | | | | | |
| ≤1 | 66 | 71.21 | 25.76 | 3.03 | - | - |
| 2-3 | 170 | 61.18 | 35.29 | 3.53 | 1.09(0.56-2.11) | 0.795 |
| 4-5 | 95 | 52.63 | 42.11 | 5.26 | 1.24(0.61-2.54) | 0.556 |
| ≥6 | 25 | 60.00 | 32.00 | 8.00 | 0.99(0.34-2.90) | 0.981 |
| **Social Media Addiction** | | | | |  |  |
| No | 278 | 65.11 | 31.65 | 3.24 | - | - |
| Yes | 78 | 44.87 | 47.44 | 7.69 | 2.87(1.69-4.88) | 0.001 |
| **Social Media Usage Time (h/d)- Divorces or Widowed (N=1675)** | | | | |  | |
| ≤1 | 240 | 56.67 | 40.00 | 3.33 | - | - |
| 2-3 | 807 | 55.51 | 40.77 | 3.72 | 1.06(0.77-1.47) | 0.707 |
| 4-5 | 480 | 58.13 | 39.38 | 2.50 | 0.84(0.59-1.19) | 0.332 |
| ≥6 | 148 | 55.41 | 36.49 | 8.11 | 1.12(0.71-1.75) | 0.636 |
| **Social Media Addiction** | | |  | |  | |
| No | 1268 | 61.83 | 35.41 | 2.76 | - | - |
| Yes | 407 | 39.56 | 53.81 | 6.63 | 2.30(1.82-2.92) | <0.001 |
| **Social Media Usage Time (h/d)- Spinsterhood (N=101)** | | | | |  | |
| ≤1 | 12 | 66.67 | 33.33 | 0.00 | - | - |
| 2-3 | 47 | 53.19 | 44.68 | 2.13 | 1.71(0.33-8.96) | 0.523 |
| 4-5 | 34 | 50.00 | 38.24 | 11.76 | 3.50(0.66-18.49) | 0.140 |
| ≥6 | 8 | 62.50 | 12.50 | 25.00 | 3.00(0.37-24.17) | 0.302 |
| **Social Media Addiction** | | | | |  |  |
| No | 77 | 59.74 | 35.06 | 5.20 | - | - |
| Yes | 24 | 37.50 | 50.00 | 12.50 | 3.7(1.48-1.13) | 0.006 |
| **Depression** | | | | | | |
| **Social Media Usage Time (h/d)- Married and Living with Spouse (N=13854)** | | | | |  | |
| ≤1 | 2145 | 69.79 | 28.48 | 1.73 | - | - |
| 2-3 | 7337 | 69.79 | 28.48 | 1.73 | 1.11(0.98-1.26) | 0.094 |
| 4-5 | 3525 | 64.00 | 34.50 | 1.50 | 1.26(1.10-1.44) | 0.001 |
| ≥6 | 847 | 56.91 | 39.08 | 4.01 | 1.82(1.52-2.20) | <0.001 |
| **Social Media Addiction** | | | |  |  | |
| No | 11110 | 69.60 | 29.14 | 1.26 | - | - |
| Yes | 2744 | 49.96 | 47.59 | 2.45 | 2.50(2.28-2.75) | <0.001 |
| **Social Media Usage Time (h/d)- Married and not Living with Spouse (N=356)** | | | | | | |
| ≤1 | 66 | 62.12 | 33.33 | 4.55 | - | - |
| 2-3 | 170 | 57.64 | 41.18 | 1.18 | 0.85(0.46-1.59) | 0.618 |
| 4-5 | 95 | 51.57 | 42.11 | 6.32 | 1.11(0.57-2.19) | 0.775 |
| ≥6 | 25 | 52.00 | 40.00 | 8.00 | 1.29(0.49-3.42) | 0.603 |
| **Social Media Addiction** | | | | |  |  |
| No | 278 | 60.43 | 36.69 | 2.88 | - | - |
| Yes | 78 | 42.31 | 51.28 | 6.41 | 2.21(1.31-3.73) | 0.003 |
| **Social Media Usage Time (h/d)- Divorces or Widowed (N=1675)** | | | | |  | |
| ≤1 | 240 | 52.92 | 42.08 | 5.00 | - | - |
| 2-3 | 807 | 53.04 | 42.63 | 4.34 | 1.07(0.78-1.47) | 0.654 |
| 4-5 | 480 | 54.17 | 42.29 | 3.54 | 1.08(0.77-1.52) | 0.648 |
| ≥6 | 148 | 50.68 | 44.59 | 4.73 | 1.25(0.81-1.94) | 0.315 |
| **Social Media Addiction** | | | |  |  | |
| No | 1268 | 57.81 | 38.88 | 3.31 | - | - |
| Yes | 407 | 38.57 | 54.30 | 7.13 | 1.97(1.56-2.48) | <0.001 |
| **Social Media Usage Time (h/d)- Spinsterhood (N=101)** | | | | | | |
| ≤1 | 12 | 75.00 | 16.67 | 8.33 | - | - |
| 2-3 | 47 | 55.32 | 44.68 | 0.00 | 2.34(0.46-12.05) | 0.308 |
| 4-5 | 34 | 44.12 | 47.06 | 8.82 | 4.44(0.84-23.39) | 0.078 |
| ≥6 | 8 | 50.00 | 37.50 | 12.50 | 5.00(0.64-39.06) | 0.125 |
| **Social Media Addiction** | | | | | | |
| No | 77 | 59.74 | 35.06 | 5.19 | - | - |
| Yes | 24 | 33.33 | 62.50 | 4.17 | 2.61(1.02-6.66) | 0.045 |

**Multimedia Appendix 6.** Associations between social media usage and mental health grouped by education

| Variables | n  (N=15986) | Proportion, % | | | Adjusted Analyses | |
| --- | --- | --- | --- | --- | --- | --- |
|  |  | Low | Middle | High | OR (95%CI) | *p* |
| **Anxiety** | | | | | | |
| **Social Media Usage Time (h/d)- Senior High School and below (N=3948)** | | | | |  | |
| ≤1 | 798 | 74.81 | 23.06 | 2.13 | - | - |
| 2-3 | 2006 | 69.39 | 29.36 | 1.25 | 1.23(0.98-1.53) | 0.070 |
| 4-5 | 911 | 68.17 | 29.86 | 1.97 | 1.29(1.00-1.65) | 0.050 |
| ≥6 | 233 | 56.65 | 36.91 | 6.44 | 2.58(1.85-3.60) | <0.001 |
| **Social Media Addiction** | | | | |  |  |
| No | 3272 | 73.07 | 25.40 | 1.53 | - | - |
| Yes | 676 | 51.92 | 44.38 | 3.70 | 2.95(2.46-3.55) | <0.001 |
| **Social Media Usage Time (h/d)- Junior College(N=6405)** | | | | |  | |
| ≤1 | 955 | 69.95 | 28.48 | 1.57 | - | - |
| 2-3 | 3410 | 68.65 | 29.79 | 1.55 | 1.00(0.84-1.20) | 0.984 |
| 4-5 | 1665 | 66.43 | 31.77 | 1.80 | 1.11(0.91-1.36) | 0.300 |
| ≥6 | 375 | 65.60 | 30.40 | 4.00 | 1.30(0.98-1.74) | 0.070 |
| **Social Media Addiction** | | | | |  |  |
| No | 5108 | 72.40 | 26.10 | 1.50 | - | - |
| Yes | 1297 | 51.11 | 46.11 | 2.78 | 2.56(2.23-2.93) | <0.001 |
| **Social Media Usage Time (h/d)- University and above(N=5633)** | | | | |  | |
| ≤1 | 710 | 71.83 | 26.06 | 2.11 | - | - |
| 2-3 | 2945 | 66.04 | 32.33 | 1.63 | 1.05(0.86-1.29) | 0.635 |
| 4-5 | 1558 | 63.93 | 34.79 | 1.28 | 1.09(0.87-1.36) | 0.461 |
| ≥6 | 420 | 56.67 | 39.05 | 4.29 | 1.62(1.23-2.15) | <0.001 |
| **Social Media Addiction** | | |  | |  | |
| No | 4353 | 70.46 | 28.37 | 1.17 | - | - |
| Yes | 1280 | 48.59 | 47.50 | 3.91 | 2.87(2.50-3.30) | <0.001 |
| **Depression** | | | | | | |
| **Social Media Usage Time (h/d)- Senior High School and below (N=3948)** | | | | |  | |
| ≤1 | 798 | 68.55 | 29.45 | 2.00 | - | - |
| 2-3 | 2006 | 64.20 | 34.30 | 1.50 | 1.14(0.93-1.40) | 0.219 |
| 4-5 | 911 | 63.89 | 34.14 | 1.97 | 1.26(1.00-1.59) | 0.052 |
| ≥6 | 233 | 51.07 | 43.78 | 5.15 | 2.36(1.71-3.25) | <0.001 |
| **Social Media Addiction** | | | |  |  | |
| No | 3272 | 67.48 | 30.84 | 1.68 | - | - |
| Yes | 676 | 48.52 | 48.37 | 3.11 | 2.23(1.87-2.68) | <0.001 |
| **Social Media Usage Time (h/d)- Junior College(N=6405)** | | | | |  | |
| ≤1 | 955 | 67.23 | 30.47 | 2.30 | - | - |
| 2-3 | 3410 | 64.57 | 34.08 | 1.35 | 1.05(0.88-1.26) | 0.212 |
| 4-5 | 1665 | 61.62 | 36.28 | 2.10 | 1.22(1.00-1.48) | 0.046 |
| ≥6 | 375 | 59.47 | 36.80 | 3.73 | 1.37(1.03-1.81) | 0.029 |
| **Social Media Addiction** | | | | |  |  |
| No | 5108 | 67.72 | 30.58 | 1.70 | - | - |
| Yes | 1297 | 48.88 | 48.80 | 2.31 | 2.41(2.11-2.76) | <0.001 |
| **Social Media Usage Time (h/d)- University and above(N=5633)** | | | | |  | |
| ≤1 | 710 | 68.31 | 29.58 | 2.11 | - | - |
| 2-3 | 2945 | 65.53 | 32.97 | 1.49 | 1.14(0.93-1.57) | 0.156 |
| 4-5 | 1558 | 62.39 | 35.94 | 1.67 | 1.30(1.04-1.63) | 0.022 |
| ≥6 | 420 | 55.24 | 40.48 | 4.29 | 1.89(1.42-2.51) | <0.001 |
| **Social Media Addiction** | | | |  |  | |
| No | 4353 | 69.19 | 29.61 | 1.19 | - | - |
| Yes | 1280 | 47.42 | 48.59 | 3.98 | 2.65(2.30-3.04) | <0.001 |

**Multimedia Appendix 7.** Associations between social media usage and mental health grouped by income

| Variables | n  (N=15986) | Proportion, % | | | Adjusted Analyses | |
| --- | --- | --- | --- | --- | --- | --- |
|  |  | Low | Middle | High | OR (95%CI) | *p* |
| **Anxiety** | | | | | | |
| **Social Media Usage Time (h/d)- Income ≤ 6000 (N=2922)** | | | | |  | |
| ≤1 | 514 | 73.74 | 24.12 | 2.14 | - | - |
| 2-3 | 1496 | 67.18 | 30.88 | 1.94 | 1.17(0.90-1.52) | 0.245 |
| 4-5 | 729 | 64.88 | 32.92 | 2.20 | 1.35(1.02-1.80) | 0.039 |
| ≥6 | 183 | 57.38 | 33.88 | 8.74 | 2.51(1.71-3.67) | <0.001 |
| **Social Media Addiction** | | | | |  |  |
| No | 2331 | 71.73 | 26.25 | 2.02 | - | - |
| Yes | 591 | 49.07 | 46.70 | 4.23 | 2.86(2.34-3.49) | <0.001 |
| **Social Media Usage Time (h/d)- Income 6001-9000 (N=3395)** | | | | |  | |
| ≤1 | 535 | 69.35 | 28.03 | 2.62 | - | - |
| 2-3 | 1814 | 65.66 | 32.91 | 1.43 | 1.09(0.86-1.38) | 0.479 |
| 4-5 | 841 | 62.31 | 35.79 | 1.90 | 1.12(0.86-1.46) | 0.402 |
| ≥6 | 205 | 57.07 | 37.56 | 5.37 | 1.79(1.25-2.57) | 0.002 |
| **Social Media Addiction** | | | | |  |  |
| No | 1686 | 69.51 | 29.00 | 1.49 | - | - |
| Yes | 709 | 47.39 | 48.80 | 3.81 | 2.64(2.21-3.17) | <0.001 |
| **Social Media Usage Time (h/d)- Income ≤ 9001 (N=9669)** | | | | |  | |
| ≤1 | 1414 | 72.49 | 25.95 | 1.56 | - | - |
| 2-3 | 5051 | 68.94 | 29.66 | 1.40 | 1.06(0.91-1.24) | 0.425 |
| 4-5 | 2564 | 67.32 | 31.28 | 1.40 | 1.13(0.96-1.34) | 0.143 |
| ≥6 | 640 | 61.56 | 35.16 | 3.28 | 1.50(1.20-1.88) | <0.001 |
| **Social Media Addiction** | | |  | |  | |
| No | 7716 | 72.80 | 26.02 | 1.18 | - | - |
| Yes | 1953 | 51.72 | 45.26 | 3.02 | 2.79(2.49-3.12) | <0.001 |
| **Depression** | | | | | | |
| **Social Media Usage Time (h/d)- Income ≤ 6000 (N=2922)** | | | | |  | |
| ≤1 | 514 | 69.85 | 27.43 | 2.72 | - | - |
| 2-3 | 1496 | 65.78 | 32.55 | 1.67 | 1.21(0.93-1.57) | 0.156 |
| 4-5 | 729 | 60.77 | 36.76 | 2.47 | 1.53(1.15-2.03) | 0.004 |
| ≥6 | 183 | 51.91 | 42.08 | 6.01 | 2.60(1.78-3.81) | <0.001 |
| **Social Media Addiction** | | | |  |  | |
| No | 2331 | 68.47 | 29.64 | 1.89 | - | - |
| Yes | 591 | 48.22 | 47.72 | 4.06 | 2.46(2.02-3.00) | <0.001 |
| **Social Media Usage Time (h/d)- Income 6001-9000 (N=3395)** | | | | |  | |
| ≤1 | 535 | 65.05 | 32.52 | 2.43 | - | - |
| 2-3 | 1814 | 61.80 | 36.88 | 1.32 | 1.07(0.85-1.34) | 0.574 |
| 4-5 | 841 | 59.57 | 38.41 | 2.02 | 1.19(0.92-1.54) | 0.175 |
| ≥6 | 205 | 54.15 | 42.93 | 2.93 | 1.61(1.13-2.29) | 0.009 |
| **Social Media Addiction** | | | | |  |  |
| No | 1686 | 65.79 | 32.69 | 1.52 | - | - |
| Yes | 709 | 44.29 | 53.03 | 2.68 | 2.29(1.92-2.74) | <0.001 |
| **Social Media Usage Time (h/d)- Income ≥ 9001 (N=9669)** | | | | |  | |
| ≤1 | 1414 | 68.39 | 29.77 | 1.84 | - | - |
| 2-3 | 5051 | 65.63 | 32.96 | 1.41 | 1.07(0.92-1.25) | 0.350 |
| 4-5 | 2564 | 63.80 | 34.48 | 1.72 | 1.20(1.02-1.42) | 0.026 |
| ≥6 | 640 | 57.50 | 38.28 | 4.22 | 1.62(1.30-2.02) | <0.001 |
| **Social Media Addiction** | | | |  |  | |
| No | 7716 | 68.90 | 29.69 | 1.41 | - | - |
| Yes | 1953 | 49.67 | 47.31 | 3.02 | 2.49(2.23-2.71) | <0.001 |

**Multimedia Appendix 8.** Associations between social media usage and mental health grouped by urban/rural

| Variables | n  (N=15986) | Proportion, % | | | Adjusted Analyses | |
| --- | --- | --- | --- | --- | --- | --- |
|  |  | Low | Middle | High | OR (95%CI) | *p* |
| **Anxiety** | | | | | | |
| **Social Media Usage Time (h/d)- Urban (N=10833)** | | | | |  |  |
| ≤1 | 1593 | 72.88 | 25.24 | 1.88 | - | - |
| 2-3 | 5681 | 67.44 | 31.05 | 1.51 | 1.11(0.96-1.28) | 0.154 |
| 4-5 | 2855 | 64.73 | 33.49 | 1.79 | 1.22(1.04-1.42) | 0.014 |
| ≥6 | 704 | 58.95 | 36.22 | 4.83 | 1.82(1.48-2.24) | <0.001 |
| **Social Media Addiction** | | | |  |  |  |
| No | 8602 | 71.66 | 26.96 | 1.38 | - | - |
| Yes | 2231 | 48.90 | 47.42 | 3.68 | 2.79(2.52-3.10) | <0.001 |
| **Social Media Usage Time (h/d)- Rural (N=5153)** | | | | |  |  |
| ≤1 | 870 | 70.57 | 27.47 | 1.95 | - | - |
| 2-3 | 2680 | 68.92 | 29.59 | 1.49 | 1.05(0.86-1.28) | 0.628 |
| 4-5 | 1279 | 68.41 | 30.26 | 1.33 | 1.06(0.85-1.32) | 0.593 |
| ≥6 | 324 | 62.04 | 33.64 | 4.32 | 1.51(1.11-2.04) | 0.008 |
| **Social Media Addiction** | | | | |  |  |
| No | 4131 | 72.43 | 26.14 | 1.43 | - | - |
| Yes | 1022 | 53.33 | 43.84 | 2.84 | 2.70(2.32-3.15) | <0.001 |
| **Depression** | | | | | | |
| **Social Media Usage Time (h/d)- Urban (N=10833)** | | | | |  |  |
| ≤1 | 1593 | 68.36 | 29.63 | 2.01 | - | - |
| 2-3 | 5681 | 64.64 | 33.99 | 1.37 | 1.14(0.99-1.31) | 0.069 |
| 4-5 | 2855 | 61.79 | 36.32 | 1.89 | 1.32(1.13-1.53) | <0.001 |
| ≥6 | 704 | 54.97 | 40.06 | 4.97 | 1.83(1.49-2.25) | <0.001 |
| **Social Media Addiction** | | | | |  |  |
| No | 8602 | 67.98 | 30.56 | 1.46 | - | - |
| Yes | 2231 | 47.74 | 48.99 | 3.27 | 2.46(2.22-2.72) | <0.001 |
| **Social Media Usage Time (h/d)- Rural (N=5153)** | | | | |  |  |
| ≤1 | 870 | 67.24 | 30.34 | 2.41 | - | - |
| 2-3 | 2680 | 65.22 | 33.21 | 1.57 | 1.02(0.85-1.24) | 0.814 |
| 4-5 | 1279 | 63.80 | 34.25 | 1.95 | 1.13(0.91-1.39) | 0.264 |
| ≥6 | 324 | 57.72 | 39.51 | 2.78 | 1.62(1.21-2.17) | 0.001 |
| **Social Media Addiction** | | | | |  |  |
| No | 4131 | 68.55 | 29.80 | 1.65 | - | - |
| Yes | 1022 | 49.31 | 47.85 | 2.84 | 2.40(2.06-2.79) | <0.001 |

**Multimedia Appendix** **9**. Associations between social media usage and mental health grouped by living status

| Variables | n  (N=15986) | Proportion, % | | | Adjusted Analyses | |
| --- | --- | --- | --- | --- | --- | --- |
|  |  | Low | Middle | High | OR (95%CI) | *p* |
| **Anxiety** | | | | | | |
| **Social Media Usage Time (h/d)- Live Alone (N=1167)** | | | | |  |  |
| ≤1 | 174 | 54.60 | 43.10 | 2.30 | - | - |
| 2-3 | 541 | 56.38 | 40.30 | 3.32 | 0.98(0.67-1.42) | 0.901 |
| 4-5 | 337 | 55.49 | 40.65 | 3.86 | 0.89(0.60-1.34) | 0.587 |
| ≥6 | 115 | 59.13 | 31.30 | 9.57 | 0.98(0.59-1.65) | 0.954 |
| **Social Media Addiction** | | | |  |  |  |
| No | 863 | 60.60 | 35.92 | 3.48 | - | - |
| Yes | 304 | 43.42 | 51.32 | 5.26 | 1.97(1.49-2.60) | <0.001 |
| **Social Media Usage Time (h/d)- not Live Alone (N=14819)** | | | | |  |  |
| ≤1 | 2289 | 73.39 | 24.73 | 1.88 | - | - |
| 2-3 | 7820 | 68.71 | 29.91 | 1.38 | 1.11(0.98-1.25) | 0.103 |
| 4-5 | 3797 | 66.79 | 31.76 | 1.45 | 1.19(1.04-1.36) | 0.011 |
| ≥6 | 913 | 60.02 | 35.93 | 4.05 | 1.80(1.50-2.15) | <0.001 |
| **Social Media Addiction** | | | | |  |  |
| No | 11870 | 72.73 | 26.02 | 1.25 | - | - |
| Yes | 2949 | 51.00 | 45.78 | 3.22 | 2.84(2.59-3.11) | <0.001 |
| **Depression** | | | | | | |
| **Social Media Usage Time (h/d)- Live Alone (N=1167)** | | | | |  |  |
| ≤1 | 174 | 54.02 | 41.38 | 4.60 | - | - |
| 2-3 | 541 | 51.20 | 44.36 | 4.44 | 1.19(0.82-1.72) | 0.359 |
| 4-5 | 337 | 49.55 | 45.40 | 5.05 | 1.23(0.83-1.83) | 0.301 |
| ≥6 | 115 | 51.30 | 41.74 | 6.96 | 1.25(0.76-2.07) | 0.382 |
| **Social Media Addiction** | | | | |  |  |
| No | 863 | 55.85 | 39.98 | 4.17 | - | - |
| Yes | 304 | 37.83 | 55.26 | 6.91 | 1.90(1.45-2.49) | <0.001 |
| **Social Media Usage Time (h/d)- not Live Alone (N=14819)** | | | | |  |  |
| ≤1 | 2289 | 69.03 | 29.01 | 1.97 | - | - |
| 2-3 | 7820 | 65.77 | 33.01 | 1.23 | 1.09(0.97-1.23) | 0.136 |
| 4-5 | 3797 | 63.55 | 34.82 | 1.63 | 1.24(1.09-1.42) | 0.001 |
| ≥6 | 913 | 56.41 | 39.65 | 3.94 | 1.79(1.50-2.14) | <0.001 |
| **Social Media Addiction** | | | | |  |  |
| No | 11870 | 69.06 | 29.61 | 1.33 | - | - |
| Yes | 2949 | 49.30 | 47.95 | 2.75 | 2.48(2.26-2.71) | <0.001 |
